# Dermoid Cysts of the Floor of the Mouth: Two Case Reports

**DOI:** 10.1155/2011/362170

**Published:** 2011-09-14

**Authors:** Christos Makos, George Noussios, Marinos Peios, Spyridon Gougousis, Pantelis Chouridis

**Affiliations:** ^1^Department of Oral and Maxillofacial Surgery, General Hospital of Kilkis, 61100 Kilkis, Greece; ^2^Department of Physical Education and Sports Sciences (at Serres), Aristotelian University of Thessaloniki, 62100 Serres, Greece; ^3^Department of Otorhinolaryngology, Head and Neck Surgery, Hippokration General Hospital, 54642 Thessaloniki, Greece

## Abstract

Dermoid cysts in the floor of the mouth may be congenital or acquired. The congenital form, according to the main theory, originates from embryonic cells of the 1st and 2nd branchial arch. The acquired form may be due to traumatic or iatrogenic causes and as a result of the occlusion of a sebaceous gland duct. Its occurrence is less and is estimated to be from 1.6 to 6.4% of the dermoid cysts of the body in adults and 0.29% of the head and neck tumors occurring in children. They may also be classified as anatomical and histological. Anatomically, they are divided into median genioglossal, median geniohyoid, and lateral cysts, while histologically they are divided into epidermoid, dermoid cysts and teratomas. Clinically, a distinction between supra and inferior type as well as between central and lateral type is proposed in relation to themylohyoidmuscle and themidline, respectively. Histologically, an estimation of dermoid, epidermoid, and teratoid cysts is reported. Enucleation via intraoral and/or extraoral approach is the method of treatment. Two case reports of dermoid cysts in the floor of the mouth are presented in this paper, and an evaluation with regard to pathology, clinical findings, differential diagnosis, and treatment is discussed.

## 1. Introduction

The term “dermoid cyst” in the floor of the mouth is used to describe three types of histologically related cysts: dermoid, epidermoid, and teratomata/teratoid cysts [1, 2]. Dermoid cysts in the floor of the mouth have been the subject of a good many researches. Jourdain (1778) called them sublingual dermoid cysts. Roser (1859) maintained that many cases of ranula and sebaceous cysts on the base of the tongue were dermoid cysts. Bytlin (1885) was preoccupied with their description but also not only with their differentiation from other similar lesions of this area, while Chairi (1891) proposed that the tumors in the floor of the mouth originated from the entrapment of epidermoid cells at the embryonic age [1, 3]. 

Dermoid cysts occur primarily in sites where embryonic parts fuse together. The majority of reported cases are in the midline of the body and especially in testis and ovaries. In the head and neck region, they occur most frequently in the periorbital area, while 6.5% of dermoid cysts in this region present in the oral cavity [4]. The incidence of floor of the mouth cysts is quite less [5]. In a study carried out by Mayo Clinic (1937), in a total of 1495 dermoid cysts of the body, 103 (6.9%) concerned the head and neck and only 24 (1.6%) the floor of the mouth [6–9], even though these findings have been challenged on account of the high (44%) rate of hair follicle cysts [2, 6]. In another study [10], of a total of 541 evident dermoid cysts of the body, 184 (34%) occurred in the head and neck and 35 (6.5%) of these in the floor of the mouth. Lastly, in yet another study, out of 1007 tumors of the head and neck area in children, 95 (9.4%) were dermoid cysts and only 3 (0.3%) occurred in the floor of the mouth. 

The present paper deals with two cases of dermoid cysts in the floor of the mouth and at the same time discusses the pathology, clinical picture, and treatment as well as the differentiation of these cysts within a broad spectrum of oncomorphous lesions in this area. 

## 2. Materials and Methods

### 2.1. Case Report 1

**Figure 1:**
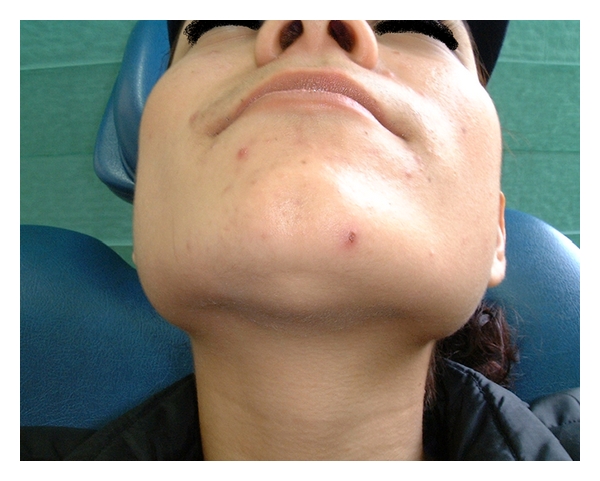
The semihard, painless, slowly evolving augmentation in the submental area.

A 21-year-old female patient came to the consultation unit of the maxillafacial surgery department to be examined for a semihard, painless, slowly evolving submental augmentation which had appeared 5 years before (Figure 1). The intraoral examination showed a nodular mass in the floor of the mouth with some floating in palpation. Extraorally, there was a small swelling in the submental region with the skin intact. The young girl complained of a progressive dysphagia and dysarthria during the last 6 months, regardless of the previous small mass in the area.

**Figure 2:**
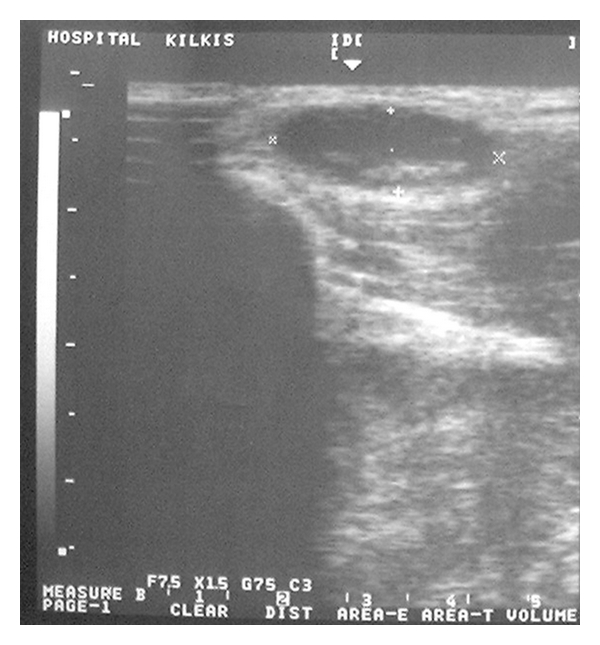
The (ECHO) ultrasonic scan of the area showing a sizeable infrasonic formation with distinct boundaries.

**Figure 3:**
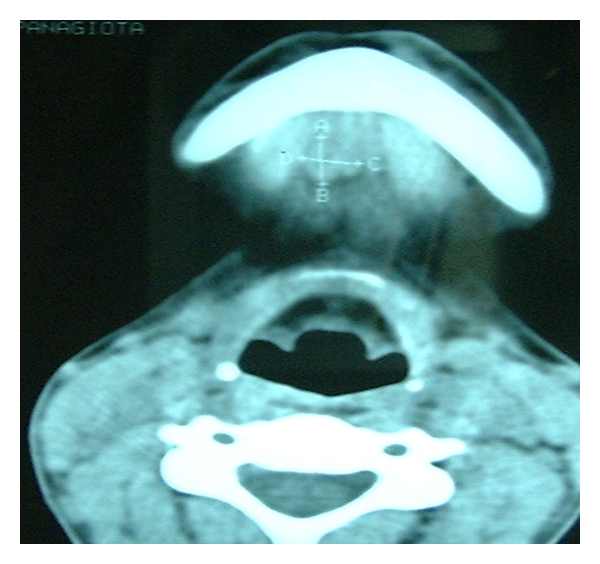
The computed tomography (CT) of the submental area showing a semitransparent cystic formation with distinct boundaries.

**Figure 4:**
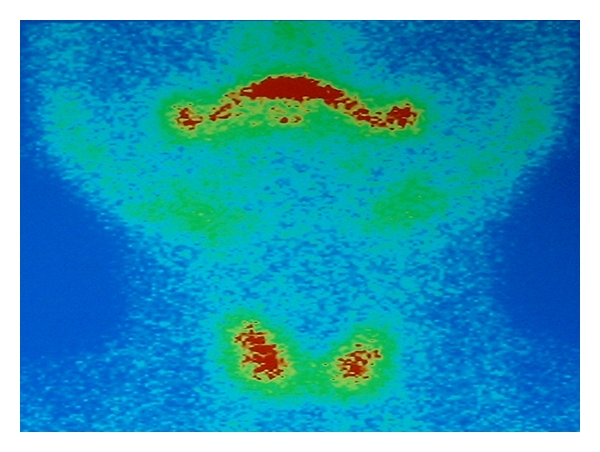
The scintiscan of the area with a Tc^99m^ showing no evidence of the existence of ectopic thyroid tissue.

The (ECHO) ultrasonic scan showed an infrasonic formation with distinct boundaries (Figure 2). The computed tomography (CT) showed a semitransparent cystic formation with distinct boundaries (Figure 3) while the scintiscan of the area with a Tc^99m^ showed no evidence of the existence of ectopic thyroid tissue (Figure 4). 

**Figure 5:**
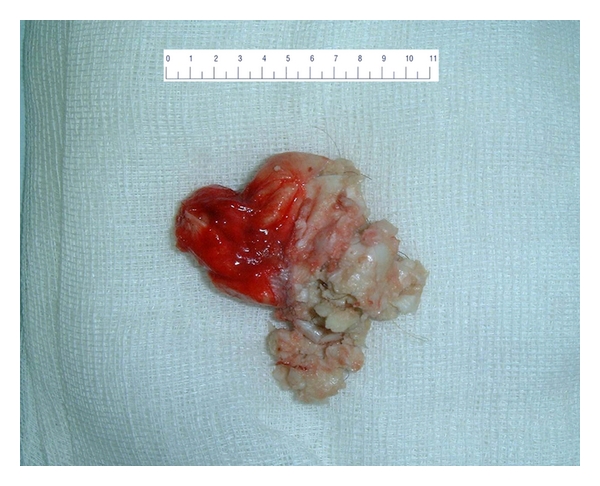
The macroscopic image of the surgical l extract with clear evidence of cystic formation (6,5 cm) containing smegma and hairs, indicative of a dermoid cyst.

**Figure 6:**
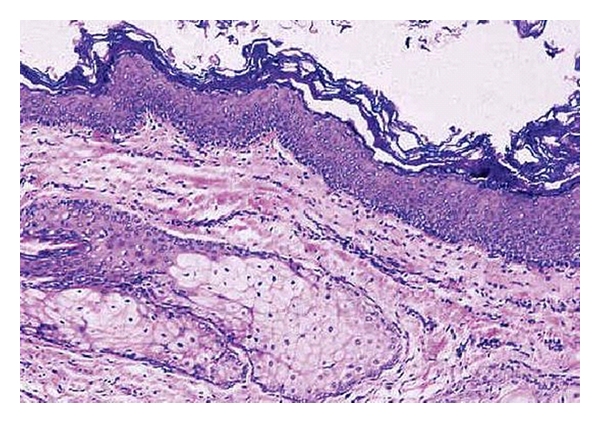
Cyst with keratinized squamous epithelium and adnexal structures in wall.

The patient was subjected to surgery, whereby the removal of the cyst was effected under general anesthesia and via submental approach. Macroscopically, the cyst (6,5 cm long) was found to contain smegma and hairs, (Figure 5) indicative of a dermoid cyst, confirmed by a pathoanatomical examination which showed a cystic capsule lined by keratinized stratified squamous epithelium and skin glands attached (Figure 6).

### 2.2. Case Report 2

**Figure 7:**
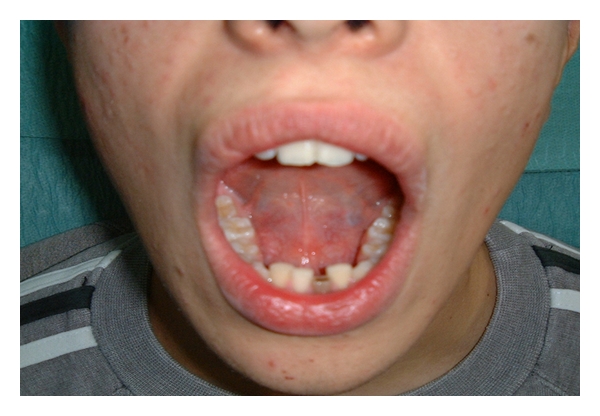
The augmentation in the floor of the mouth due to the intraoral projection of the augmentation.

A 17-year-old male patient came to the consultation unit of the maxillofacial surgery department to be examined for an inflamed, painful, and considerably large augmentation of the submaxillary area which had created a swelling in the floor of the mouth (Figure 7) and had appeared 2 years before. 

The intraoral examination showed a round mass which slightly elevated the tongue. Extraorally there was a painful swelling in the submaxillary area which did not disappear when he opened his mouth.

**Figure 8:**
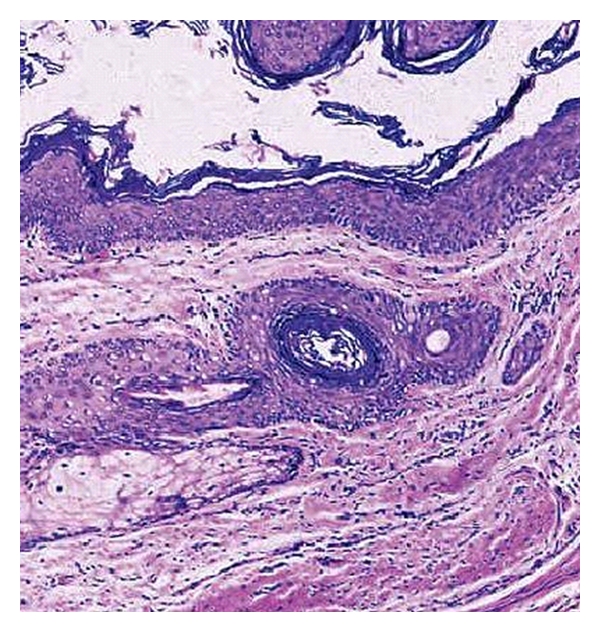
Keratinizing squamous epithelium with distinct granular layer with a sebaceous gland in the cyst wall.

The histopathological examination showed a cyst with keratinization in the overlying epithelium, with the presence of a sebaceous gland in the wall (Figure 8). 

**Figure 9:**
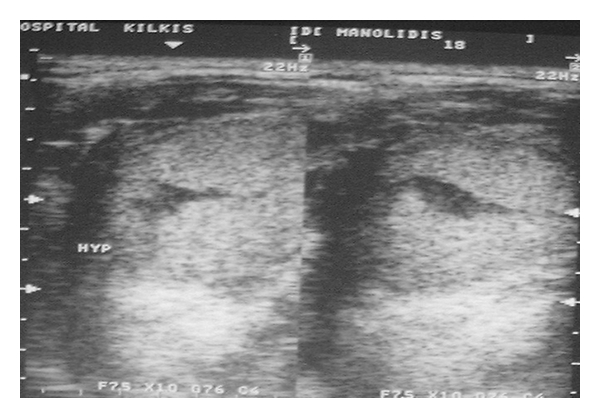
The (ECHO) ultrasonic scan of the area showing a sizeable infrasonic formation with distinct boundaries.

**Figure 10:**
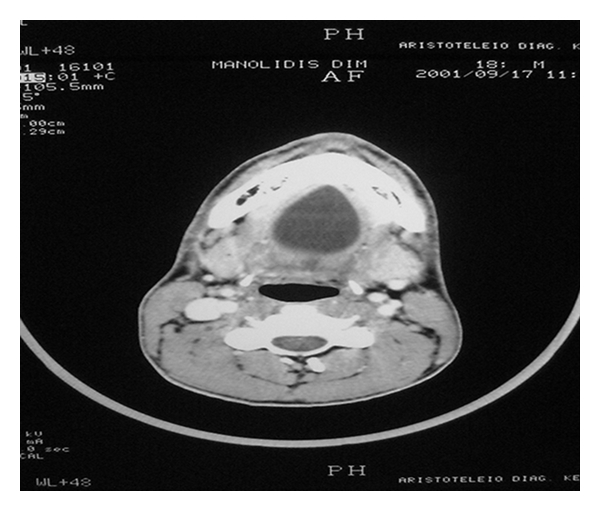
Section of computed tomography (CT) showing a sizeable cystic formation with distinct boundaries.

**Figure 11:**
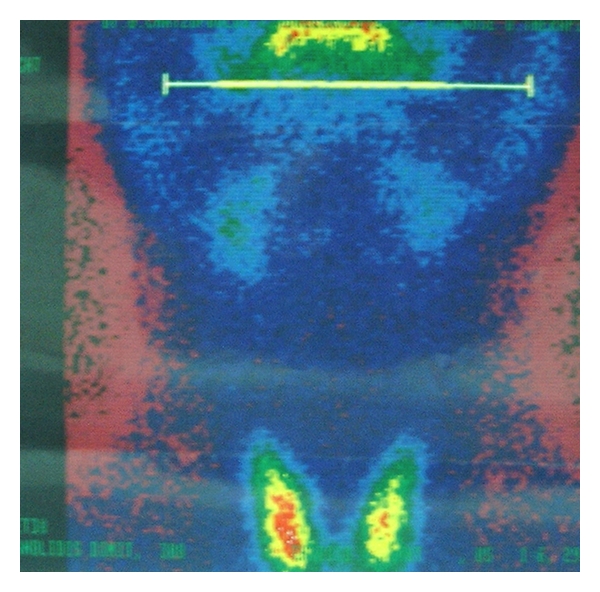
The scintiscan of the area with a Tc^99m^ showing no evidence of the existence of ectopic thyroid tissue.

The (ECHO) ultrasonic scan of the area (Figure 9) showed a sizeable infrasonic formation with distinct boundaries. The computed tomography (CT) showed a sizeable cystic formation with distinct boundaries (Figure 10), while the scintiscan of the area with a Tc^99m^ showed no evidence of the existence of ectopic thyroid tissue (Figure 11). 

**Figure 12:**
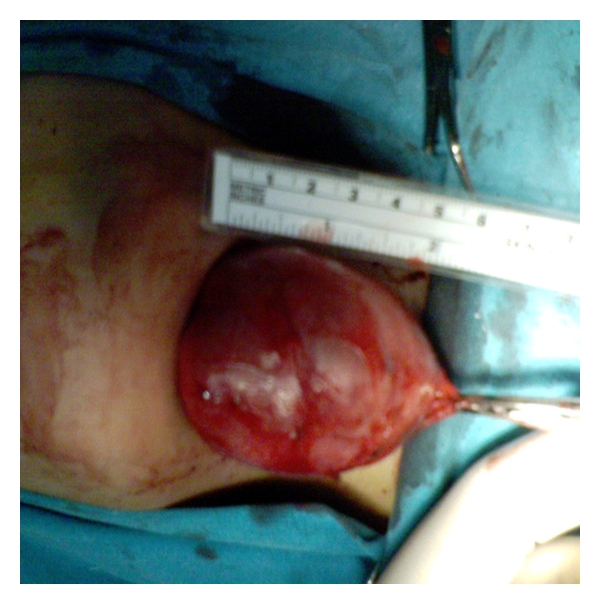
The macroscopic image of the surgical extract showing the cystic formation (5,4 cm) of our second case.

The clinical findings bore evidence of a cyst in the floor of the mouth with an infection which was surgically treated via intraoral approach, removing a 5.4 cm long cyst (Figure 12). By virtue of the infection of the cyst and the surrounding anatomical elements, ampicillin antibiotic was administered per os at a dosage of 500 mg × 4 for 7 days. 

## 3. Discussion

The term “dermoid cyst” in the floor of the mouth is used to describe three types of histologically related cysts in this area: dermoid, epidermoid, and teratomata/teratoid cysts [1, 2]. Dermoid cysts in the floor of the mouth have been the subject of a considerable number of researches. Dermoid cysts may be classified into 2 major categories: congenital and acquired forms. 

In international bibliography, reference is made to 3 theories with regard to the origin of cysts in the floor of the mouth. According to the 1st and most prevalent theory, these cysts originate from embryonic cells of the 1st and 2nd branchial arch entrapped in the mesenchyme of the area during the 3rd/4th week of embryonic life. With regard to the 2nd theory, it explains the pathogenic mechanism of the acquired form. The acquired cysts may be due to the implantation of epithelial cells subsequent to accidental or surgical injury (traumatic causes, iatrogenic antecedents, or an occlusion of a sebaceous gland duct). 

Lastly, the 3rd theory maintains that these cysts are considered a variation of the cyst of the thyroglossal pore [9–13]. 

Anatomically, it is possible to distinguish 3 different types of dermoid cysts: median genioglossal, median geniohyoid, and lateral cysts, according to the anatomic relationship between the cyst and the muscles of the floor of the mouth. So with regard to the midline, these cysts are differentially diagnosed as central and lateral cysts [7], while with relation to the mylohyoid muscle they are differentially diagnosed as supra and inferior cysts of the mylohyoid muscle [7, 14]. According to one point of view, lateral cysts are, in effect, central cysts which have undergone displacement due to the development of strong fibrous symphyses with the tissues of the midline. As far as another opinion is concerned, the lateral cysts constitute a separate entity [12]. 

In histological terms, dermoid cysts in the floor of the mouth are categorized according to Meyer's classification [11], separating them into dermoid, epidermoid, and teratoid/teratomata cysts. Dermoid cysts have a wall of stratified squamous cornified epithelium and contain smegma and keratic scales as well as cutaneous appendages such as hair follicles, hairs, and sebaceous and sudoriparous glands. Epidermoid cysts have a wall of stratified squamous usually cornified epithelium and contain smegma and keratic scales without cutaneous appendages. Lastly, teratoid/teratomata cysts have a wall of stratified squamous epithelium with or without cornification and contain smegma and keratic scales as well as elements of the middle blastoderm such as vascular formations, elements of muscle and bone, dental tissues, or even whole teeth [1, 8]. The latter type is the only variety that may have a malignant transformation. The clinical findings of these cysts cannot be differentially diagnosed. 

Epidermoid cysts are less common than dermoid cysts in the head and neck region.

Most patients with these lesions ranged between the 2nd and 3rd decades of life. There is no predominant sex in the recent literature, regardless of some evidence which showed a male prevalence [4]. These cysts generally appear in the floor of the mouth, where there is a firm, usually painless, swelling, which sometimes raises the tongue. There are also symptoms of dysphagia and dysarthria and, when the lesions are quite large, dyspnea. A double-chin appearance is also common if the cyst develops below the mylohyoid muscle.

Depending on the position of its development and the size of the cyst, it either manifests itself as an augmentation in the submental area or as an augmentation in the floor of the mouth of a usually dough-like constitution and a double chin. In the case of a sizeable cyst, there is discomfort in chewing and in speech (croaking) or even in breathing, while in the event of infection there is rubor and pain in the area [1, 9, 11, 14]. The appearance of these cysts is most frequently reported in adults in their 20' s and 30' s [1, 9, 14]. 

Of considerable assistance in the diagnosis of cysts in the floor of the mouth are the (ECHO) ultrasonic scan, the computed tomography (CT), the magnetic resonance imaging (MRI), and the scintiscan of the area [2, 7]. In the ultrasonic scan (ECHO), these cysts appear as infrasonic areas with ultrasonic elements; the computed tomography and magnetic resonance imaging specify their size and anatomical relationship, while the scintiscan helps in their differential diagnosis from the ectopic thyroid tissue. The injection of endographine or lipiodol could assist in defining the boundaries and the size of the cysts [1]. Nowadays, however, paracentesis of these cysts is usually avoided owing to their viscous content as well as the risk of their infection [15].

The cysts in the floor of the mouth are differentially diagnosed with relation to a large number of diseases which occur in this area with similar symptomatology. Differential diagnosis should include developmental lesions, congenital, inflammatory, and salivary gland lesions, lymphomas, and benign tumors. 

Developmental lesions are branchial cleft cyst, heterotopic gastrointestinal cysts, thyroglossal duct cysts, and ectopic thyroid tissue. Congenital masses include vascular malformations and lymphangiomas. Inflammatory accumulations are the Ludwig angina, cellulitis, and submandibular and sublingual space infections. Salivary gland deformities are ranula and the obstruction and dilation of the Wharton pore, cysts of the thyroid pore, pleomorphic adenoma, and mucoepidermoid carcinoma. Lymphomas are considerable in the area and coexist with lymphadenopathy. They are usually MALT lymphomas of low-grade extranodal B cells and often associated with autoimmune disease. Benign tumors are also rare and are of mesenchmal origin such as lipoma, schwannoma, and leiomyoma [9, 13, 16]. Needless to say, in all cases the conclusive confirmation of their diagnosis is effected via histopathological examination, as mentioned above [9, 13]. 

Finally, the chosen treatment for cysts in the floor of the mouth is their total extraction (enucleation) via intraoral or extraoral approach or a combination of both, determined on each occasion by the location and size of the cyst [1, 6, 9, 14]. There are no specific rules for treating these lesions because they are mainly congenital. When the symptoms include dysphagia and dyspnea, the doctor should guide the patient to undergo an operation. In most cases, the enucleation can be carried out intraorally, as clearly evident in a review of international bibliography, whereby in 194 cases of cysts of which 120 were surgically treated, 70 (58%) were done intraorally, 37 (31%) extraorally, and 13 (11%) via a combination of intra- and extraoral approaches [7, 10]. As regards location, out of 58 cases of sublingual approaches there are 5 reported cases of extraoral approach and 6 cases of combined approach; of 58 cases of submental approach, there are reports of 20 cases treated extraorally, 7 cases treated intraorally, and 2 treated with the combination of the intra- and extraoral approaches, while of 4 cases of submental approach, 3 cases were done via intraoral approach and 1 via extraoral approach [7, 10].

In our 2 case reports, Case  1 was treated via submental approach while Case  2 was done via intraoral approach. In Case  1, the surgical intervention yielded a dermoid cyst 6.5 cm long while in Case  2 it yielded a 5.4 cm long cyst. In both cases, the patients were observed for 2 years (at 6-month intervals) during which time there were no pathological findings. Prognosis is very good, and recurrence rate is very low and is usually related to bone remnant of the genial tubercles or of the hyoid bone [17].

## 4. Conclusion

In conclusion, dermoid cysts in the floor of the mouth are quite rare and need to be differentially diagnosed from several other diseases and conditions of the area. For their diagnosis, their clinical picture is essential involving a detailed examination of their size and anatomical location. Furthermore, invaluable assistance is provided by the ultrasonic scan, computed tomography, and scintiscan of the area. Their treatment calls for careful planning and execution of the surgical operation. The ultimate confirmation and definite diagnosis of the disease is always effected via histopathological examination.

## References

[1] Gold BD, Sheinkopf DE, Levy B (1974). Dermoid, epidermoid and teratomatous cysts of the tongue and the floor of the mouth. *Journal of Oral Surgery*.

[2] Kinzer S, Mattern D, Ridder GJ (2006). Diagnostic and therapeutic management of a big cyst at the floor of the mouth—a case report. *Laryngo-Rhino-Otologie*.

[3] Ohishi M, Ishii T (1985). Dermoid cyst of the floor of the mouth: lateral teratoid cyst with sinus tract in an infant. *Oral Surgery, Oral Medicine and Oral Pathology*.

[4] Bonet-Coloma C, Mínguez-Martínez I, Palma-Carrió C, Ortega-Sánchez B, Peñarrocha-Diago M, Mínguez-Sanz JM (2011). Orofacial dermoid cysts in pediatric patients: a review of 8 cases. *Medicina Oral, Patologia Oral y Cirugia Bucal*.

[5] Pirgousis P, Fernandes R (2011). Giant submental dermoid cysts with near total obstruction of the oral cavity: report of 2 cases. *Journal of Oral and Maxillofacial Surgery*.

[6] Brown CA, Baker RD (1972). Dermoid cyst of the floor of the mouth: lateral teratoid cyst with sinus tract in an infant. *Journal of Oral Surgery*.

[7] King RC, Smith BR, Burk JL (1994). Dermoid cyst in the floor of the mouth. Review of the literature and case reports. *Oral Surgery, Oral Medicine and Oral Pathology*.

[8] New BG, Erich JB (1937). Dermoidcyst of the floor of the head and neck. *Surgery Gynecology and Obstetrics*.

[9] Oatis S, Hartman GL, Robertson GR, Sugg WE (1975). Dermoid cyst of the floor of the mouth. Report of a case. *Oral Surgery*.

[10] Taylor BW, Erich JB, Dockerty MB (1960). Dermoids of the head and neck. *Minnesota Medicine*.

[11] Longo F, Maremonti P, Mangone GM, De Maria G, Califano L (2003). Midline (dermoid) cysts of the floor of the mouth: report of 16 cases and review of surgical techniques. *Plastic and Reconstructive Surgery*.

[12] Lin HW, Silver AL, Cunnane ME, Sadow PM, Kieff DA (2011). Lateral dermoid cyst of the floor of mouth: unusual radiologic and pathologic findings. *Auris Nasus Larynx*.

[13] Torres JS, Higa TT (1970). Epidermoidal cysts in the oral cavity. Report of three cases. *Oral Surgery*.

[14] Walstad WR, Solomon JM, Schow SR, Ochs MW (1998). Midline cystic lesion of the floor of the mouth. *Journal of Oral and Maxillofacial Surgery*.

[15] Masuda BJ (1946). Dermoid cyst in the floor of the mouth. *The American Journal of Orthodontics and Dentofacial Orthopedics*.

[16] Cortezzi W, De Albuquerque EB (1994). Secondarily infected epidermoid cyst in the floor of the mouth causing a life-threatening situation: report of a case. *Journal of Oral and Maxillofacial Surgery*.

[17] Kim JP, Park JJ, Jeon SY Endoscope-assisted intraoral resection of external dermoid cyst.

